# Seropositivity and risk factors associated with the presentation of bovine leukosis virus in Sotaquirá, Colombia

**DOI:** 10.14202/vetworld.2021.2212-2218

**Published:** 2021-08-26

**Authors:** Diana M. Bulla-Castañeda, Adriana M. Díaz-Anaya, Diego J. Garcia-Corredor, Julio C. Tobón-Torreglosa, Diego Ortiz Ortega, Martín O. Pulido-Medellín

**Affiliations:** 1 Grupo de Investigación en Medicina Veterinaria y Zootecnia (GIDIMEVETZ), Universidad Pedagógica y Tecnológica de Colombia (UPTC), Tunja, Colombia; 2 Doctoral Program in Biomedical and Pharmaceutical Sciences, University of Namur, Namur, Belgium; 3 Doctorado en Ciencias Biológicas y Ambientales (UPTC), Tunja, Colombia; 4 Compañía Colombiana de Productos Veterinarios (VECOL), Bogotá, Colombia; 5 Corporación Colombiana de Investigación Agropecuaria (AGROSAVIA), Mosquera, Colombia

**Keywords:** bovine, bovine illness, bovine disease, antibodies, bovine leukosis virus

## Abstract

**Background and Aim:**

Enzootic bovine leukosis is a disease economically important to the dairy farming industry worldwide. The virus is of the Deltaretrovirus genus and is primarily transmitted iatrogenically. Most bovines infected with the virus remain asymptomatic with only 5-10% of cattle having lymphomas. This study aimed to determine the seroprevalence of bovine leukosis virus (BLV) in Sotaquirá, Boyacá, Colombia.

**Materials and Methods:**

We conducted a descriptive, observational epidemiological cross-sectional study using the simple random sampling method with a sample size of 1000. Blood samples from random bovine were processed using the SERELISA^®^ BLV Ab Mono Blocking indirect enzyme-linked immunosorbent assay kit (Zoetis, USA). The assay had a sensitivity of 97% and a specificity of 98%. The collected data were processed using Epi Info^®^ (Centers for Disease Control and Prevention; Atlanta, Georgia). From the study, we could determine a high seroprevalence of BLV in Sotaquirá.

**Results:**

We established a high seroprevalence on BLV in the municipality, with 31.1% apparent seroprevalence and 30.6% real seroprevalence rate. We found that male cattle more than 4 years old (39.4%) and the Ayrshire breed (45.5%) had the highest prevalence rates of the virus. In this study, we could establish statistically significant associations according to breed, age, and gender of the cattle under study. Moreover, we identified the risk factors for BLV infection. We found that in cattle aged <1 year and those older than 4 years of age and those of the Holstein breed, the presentation of infectious bovine rhinotracheitis, mucosal secretions, mastitis, fetal death, the presence of a corral, and the implementation of artificial insemination practices were risk factors for BLV infection.

**Conclusion:**

Determining the prevalence of BLV within the herd and identifying the associated risk factors for the disease are fundamental in developing efficient programs for the control and eradication of BLV within herds.

## Introduction

Bovine leukosis virus (BLV) is a member of the *Deltaretrovirus* genus and *Retroviridae* family. The virus is etiologically associated with the presentation of enzootic bovine leukosis (EBL) [1]. BLV infection is one of the most economically important to the dairy cattle industry worldwide [2,3]. BLV is transmitted horizontally through arthropods, such as the gadfly. Calves acquire the virus *in utero* or by ingesting colostrum from another infected cow. Iatrogenic transmission of the virus can occur from using surgical tools or tubing contaminated with the blood of infected animals during rectal palpation [4]. Most cattle infected with BLV remain asymptomatic throughout their productive life. In other words, the infection remains clinically dormant in an aleukemic state [1,5]. Approximately 5-10% of cattle develop pathologies consistent with EBL, manifesting lymphoma or lymphosarcoma, predominant in animals between 3 and 5 years of age [1,4]. EBL is a disease requiring declaration in most European countries. Official methods of control include detection or monitoring of pathology, tracking movements of cattle across borders, and eradication of the animal [5,6].

Therefore, EBL remains common worldwide with a prevalence of 90% of herds in endemic areas, including East Europe, South America, and various Asian countries [7]. The high prevalence of the disease adversely affects farmers economically since the disease increases the occurrence of seizures in cattle in farms and slaughterhouses [8]. Furthermore, EBL is considered one of the five most important infectious agents in cattle, as it is fundamental for evaluating animals to obtain more complete diagnostic data about the current state of the virus [9]. In Colombia, a positive diagnosis of the disease requires immediate reporting (ICA Resolution 3714 of 2015). Recent reports have described the distribution of the disease by department. As some examples of how widespread the disease is, 69-90% of cattle in the Department of Cundinamarca are infected, 73-100% in Antioquia, and 85-100% in Meta, to name a few [9]. Despite these alarmingly high prevalence rates, no protocol or procedure exists aimed at preventing BLV in cattle while also having a significant impact in mitigating the economic burden [10].

In Boyacá, studies allowing the identification of the epidemiological status of the disease are limited [9,11], even though cattle raising in the department is one of the most important economic activities in Colombia. For these reasons, this study was designed to determine the seroprevalence of BLV in the municipality of Sotaquirá, Boyacá, and understand the risk factors that predispose cattle to BLV infection.

## Materials and Methods

### Ethical approval and Informed consent

The study was approved by the Ethic Committee of Universidad Pedagógica y Tecnológica de Colombia. This study was performed in compliance with Law 576 of the 2000 Code of Ethics in Veterinary Medicine and Law 84 of the 1989 Statue of Animal Protection of the Republic of Colombia. Informed consent was obtained from cattle owners before sample collection for this study.

### Study period and location

The study was carried out between July 2018 and July 2019, this study was conducted in the municipality of Sotaquirá, Boyacá, located in the Central Eastern region of Colombia at an altitude of 2800 m above sea level. The municipality has an average temperature of 14°C and has an extension of 288.65 km. Cattle production is done primarily in the plains regions with large extensions and in hillside regions worked on by small and midsized farms. Sotaquirá is part of the so-called “milk corridor” in the department of Boyacá and has recently increased its development in the production of meat and milk [12].

### Determination of sample size

The municipality of Sotaquirá had a cattle population of approximately 19,333 heads [13]. Keeping this population size in mind, we determined a sample size of 1000 cows with a sampling fraction of 5.17%. These values were obtained using the equation for determining the sample size for an interval estimate of a population proportion shown below.


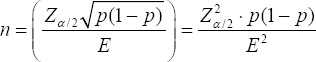


We considered an accepted error of 3.1% with a 95% confidence interval (CI) and an expected prevalence of 50% due to the lack of similar studies conducted in the region.

### Evaluated variables

We classified the variables evaluated into two broad categories: (i) Variables relating to the animals and (ii) variables at the site. For this study, we grouped the cattle under study according to breed, age, gender, EBL disease manifestation, and positivity rate of diseases of importance to cattle herding. The second category of variables was concerned with working practices in the farms that participated in the study.

### Sample collection and sampling

Blood samples were obtained by coccygeal venipuncture from cattle of different breeds. Specifically, we sampled from the Ayrshire, Holstein, Jersey, Normando, and Zebu breeds and cross-breeds. We further sampled between genders and age groups within each breed. Approximately 7 mL of blood were collected from each animal and were collected in Vacutainer tubes (Becton, Dickinson and company, USA) and refrigerated for transport to the Veterinary Parasitology Laboratory at the Universidad Pedagógica y Tecnológica de Colombia. At the laboratory, the samples were centrifuged (Hermle, Z206A, Germany) at 2500 rpm for 10 min to obtain serum. The serum was transferred to Eppendorf tubes using a Pasteur pipette and stored at −20°C, as previously described [14]. The samples were tested using indirect enzyme-linked immunosorbent assay (ELISA) using the SERELISA^®^ BLV Ab Mono Blocking commercial kit (Zoetis, USA), according to the manufacturer’s instructions. The assay has a sensitivity of 97% and a specificity of 98%.

### Statistical analysis

For the analysis of the data obtained, a descriptive, observational, cross-sectional study using the simple random sampling method was conducted. Then, the database was refined and consolidated for processing using the Epi Info^®^ statistical software package (Centers for Disease Control and Prevention; Atlanta, Georgia). Determining factors were calculated using prevalence ratio (PR). The dependent variables used were the serological results, whereas the independent variables used were all determining factors established by the structured epidemiological survey performed during sample collection. Then, these factors were used to construct the final model using logistic regression analysis.

## Results

Of the 1000 cattle, 868 were female and 132 were male. From these, the apparent seroprevalence (AP) of 31.1%, where 293 were female (33.6%) and 18 were male (14.5%), tested positive for antibodies against the virus. The real seroprevalence (RP) rate was 30.6% with a positive predictive value of 95.5%, indicating that a positive result has a 95.5% probability of identifying an infected animal. The negative predictive value was 98.7%, indicating that a negative result has 98.7% probability of identifying a healthy animal.

Regarding the breeds tested, the Ayrshire and Holstein breeds showed the highest seroprevalence with RP rates of 45.8% and 41.9%, respectively. The breeds with the highest seroprevalence rates were followed successively by the Jersey, Zebu, cross-breed, and Normando breeds. Interestingly, the Zebu breed and cross-breeds showed identical AP and RP, whereas the Normande breed showed the lowest seroprevalence with an AP of 11.3% and RP of 9.8% (Table-1).

**Table-1:** AP and RP of BLV by bovine breed in Sotaquirá, Boyacá.

Breed	n	Positive	AP (%)	RP (%)	PPV (%)	NPV (%)
Ayrshire	11	5	45.5	45.8	97.6	97.5
Holstein	601	251	41.8	41.9	97.2	97.8
Jersey	21	6	28.6	28.0	95.0	98.8
Zebu	22	4	18.2	17.1	90.9	99.4
Cross-Breed	88	16	18.2	17.1	90.9	99.4
Normando	257	29	11.3	9.8	84.0	99.7

AP=Apparent seroprevalence, RP=Real seroprevalence, PPV=Positive predictive value, NPV=Negative predictive value

Of the age groups evaluated, cattle older than 4 years showed a higher seroprevalence than any other age groups (Table-2). Moreover, we found that cattle aged 1-2 years showed the lowest seroprevalence with an AP of 21.1% and RP of 20.1% (Table-2).

**Table-2:** AP and RP of BLV by age group in bovine from the municipality of Sotaquirá, Boyacá.

Age group (years)	n	Positive	AP (%)	RP (%)	PPV (%)	NPV (%)
<1	179	42	23.5	22.6	93.4	99.1
1-2	209	44	21.1	20.1	92.4	99.2
2-4	112	28	25.0	24.2	93.9	99.0
>4	500	197	39.4	39.4	96.9	98.1

AP=Apparent seroprevalence, RP=Real seroprevalence, PPV=Positive predictive value, NPV=Negative predictive value, BLV=Bovine leukosis virus

The Holstein and Normando breeds (p<0.001) and cross-breeds (p=0.003) showed a statistically significant association in the presence of antibodies against BLV. Similarly, we found that three of the age groups – cattle <1 year old (p<0.001), those between the ages of 1 and 2 years (p<0.001), and those more than 4 years old (p<0.001) – showed a statistically significant association in the presence of antibodies against BLV. We are also able to determine an association due to gender (p<0.001) in the animals sampled, indicating that the presence of the disease is associated with breed, age, and gender in the cattle sampled (Table-3).

**Table-3:** Analysis of breed, age, and gender as a risk factor associated with BLV infection.

Variable	Category	PR	95% CI	p-value
Breed	Holstein	1.4589	1.3477-1.5793	<0.001
	Normando	0.6994	0.6513-0.751	<0.001
	Ayrshire	1.2661	0.737-2.175	0.234
	Zebu	0.8386	0.6855-0.9214	0.136
	Cross-breed	0.8269	0.7421-0.9214	0.003
	Jersey	0.9638	0.733-.2674	0,505
Age (years)	<1	1.2739	1.1698-1.3873	<0.001
	1-2	0.8391	0.77-0.9144	<0.001
	2-4	0.9084	0.8089-1.0201	0,083
	>4	1.2739	1.1698-1.3873	<0.001
Gender	-	0.7766	0.7134-0.8455	<0.001

Results are shown as prevalence ratio (PR) and 95% confidence interval (CI). Significance is denoted by p<0.05

In our analysis, we determined whether the presence of BLV is associated with other diseases relevant to cattle production. We found only infectious bovine rhinotracheitis (IBR) to have a significant association (PR=1.0049; 95% CI=0.9236-1.0933; p=0.0005) (p≤0.05) (Table-4).

**Table-4:** Analysis of infectious diseases as a risk factor associated with BLV infection.

Variable	PR	95% CI	p-value
Bovine viral diarrhea	1.0049	0.9236-1.0933	0.481
Infectious bovine rhinotracheitis	1.1529	1.0621-1.2514	0.0005
Neosporosis	0.9829	0.9041-1.0684	0.368
Paratuberculosis	1.2132	0.9078-1.6213	0.091

Results are shown as prevalence ratio (PR) and 95% confidence interval (CI). Significance is denoted by p<0.05, BLV=Bovine leukosis virus

Moreover, we analyzed the working practices instituted at the farms from which the samples were obtained. We found that the presence of corrals, cattle from various ranchers, artificial insemination, lending of cattle breeders, and pest control showed significant statistical association with the presence of antibodies against BLV, showing a relationship between field hand practices and disease propagation (Table-5).

**Table-5:** Analysis of field and practices as possible risk factors for BLV.

Variable	PR	95% CI	p-value
Presence of corral	1.2412	1.1464-1.3439	<0.001
Cattle from multiple owners	0.818	0.7551-0.8862	<0.001
Artificial insemination	1.4427	1.3374-1.5563	<0.001
Vaccination	1.0432	0.9523- 1.1427	0.197
Needle for each animal	1.0099	0.7682-1.3277	0.573
Other animal species	0.7961	0.5719-1.1082	0.081
Livestock exhibitions	1.1933	0.9227-1.5433	0.084
Breeder lending	0.8237	0.7563-0.8972	<0.001
Rodent control	1.2878	1.1905-1.3931	<0.001

Data are presented as prevalence ratio (PR) and 95% confidence interval (95% CI), BLV=Bovine leukosis virus

Similarly, we analyzed the different clinical manifestations often observed as possible risk factors for BLV infection. We found that cattle with mucosal secretions, mastitis, and fetal death were significantly associated with the presence of BLV infection in the cattle under study (Table-6).

**Table-6:** Analysis of clinical manifestations as possible risk factors associated with BLV infection.

Variable	RP	IC 95%	p-valor
Mucosal secretion	1.2454	1.1139-1.3925	<0.001
Mastitis	1.24	1.1429-1.3454	<0.001
Fever	1.0273	0.9431-1.119	0,290
Conjunctivitis	0.9605	0.8678-1.0632	0.252
Respiratory symptoms	1.0165	0.927-1.1147	0.390
Fetal death	1.283	1.1227-1.4662	<0.001

Data are presented as prevalence ratio (PR) and 95% confidence interval (95% CI), BLV=Bovine leukosis virus

Using logistic regression analysis, we could determine both risk factors for BLV and protective factors against BLV. We report these factors as the odds ratios shown in Table-7. Variables with an odds ratio >1 were considered risk factors, whereas those with an odds ratio <1 were considered protective factors. An odds ratio of 1 indicates that there is neither a positive nor negative association between the variable and BLV infection.

**Table-7:** Analysis of variables as possible risk factors associated with BLV infection.

Variable	Odds ratio	LCI (95%)	UCI (95%)	p-value
<1 year	2.2008	1.671	2.8984	<0.001
1-2 years	0.5233	0.3638	0.7529	<0.001
>4 years	2.2008	1.671	2.8984	<0.001
Holstein	4.0517	2.9457	5.573	<0.001
Normando	0.208	0.1376	0.3147	<0.001
Cross-breed	0.4648	0.2657	0.813	0.0072
Gender	0.4061	0.2417	0.6822	<0.001
IBR	1.4161	1.0633	1.8859	0.0173
Presence of corral	2.093	1.5335	2.8565	<0.001
Breeder loan	0.5523	0.3613	0.8445	0.0061
Presence of other rancher cattle	0.5678	0.4106	0.7851	<0.001
Mucosal secretion	5.4079	2.4855	11.7666	<0.001
Artificial insemination	1.4841	1.0826	2.0346	0.0142
Mastitis	1.6661	1.1997	2.3139	0.0023
Fetal death	1.4863	1.0444	2.1153	0.0277

BLV=Bovine leukosis virus, IBR=Infectious bovine rhinotracheitis

## Discussion

BLV is a disease with worldwide distribution. In Colombia, several studies have reported the prevalence of the disease. The most recent study published in 2020 by Corredor *et al*. [9] has shown the distribution of the virus to be between 69% and 90% in Cundinamarca, 71-94% in Boyacá, 73-100% in Antioquia, 85-100% in Meta, 14-75% in Nariño, and 17-75% in Cesar. Other studies have reported the seroprevalence rates in other departments across the country, such as 21% in Montería [15], 73% in Mesa de los Santos [16], 19.8% in Pasto [17], 15% in Yopal [18], and 44% in Antioquia [19].

Similarly, molecular testing has shown that the central region of the country has the highest percentage of animals living with the disease (50%), followed by the oriental region (15%), the northern region (8.2%), and the southern region (5.2%) [20]. In Boyacá, the most recent registry has found that the disease has a seroprevalence of approximately 13.5% [11], a value that is below the reported value in this study in the municipality of Sotaquirá.

Internationally, BLV has a prevalence of approximately 90.16% in Argentina [21], 10.4% in Uruguay [22], 73.3% in Japan [23], 68% in Canada [2], 5.6% in Ecuador [24], and 92.7% in Peru [25]. These data indicate that at the international level, as with the national level, the prevalence rates vary. This may be due to differences in study design and sampling and the indirect effects of other health phenomena to which cattle are susceptible, such as herd sizes and composition [26].

Considering the cattle breeds under study, the Ayrshire and Holstein breeds showed the highest seroprevalence. The reported data in this study contradict those reported in a 2008 study by Betancur and Rodas [15], who determined seroprevalence of 20.9% in European milk-producing cattle. Furthermore, Hernandez *et al*. [27] have reported a seroprevalence of 80.7% in Holstein cattle, whereas Kobayashi *et al*. [1] determined a prevalence of 47.4% in the same breed. Similarly, studies conducted in Colombia have reported a seroprevalence of 12.7% in the Zebu breed and 28.7% in cross-breeds [15], values are different from those found in Sotaquirá for both breeds (~18.2%).

Regarding the age groups used in this study, cattle older than 4 years showed a higher seroprevalence, whereas cattle aged between 1 and 2 years showed a lower seroprevalence (Table-2). Betancur and Rodas [15] have reported a higher seroprevalence in cattle older than 7 years (~30%), whereas cattle aged 3-4 years had a lower seroprevalence of approximately 11.3%. In addition, Hernandez *et al*. [27] have found that cattle older than 4 years have a high seropositivity rate at approximately 26%, whereas animals younger than 2 years had a lower seroprevalence at approximately 14%, indicating that with increasing age, the susceptibility to BLV infection increases in cattle. This can potentially be explained by an animal’s increased risk of exposure to BLV by being in closer contact with other infected cattle [15,16].

The variation in seroprevalence in these age groups could arise from sample collection: If sample collection was performed during the early phase of BLV infection, the animal had not yet undergone seroconversion [23]. BLV requires a rather long latency period to cause the pathology, which could then explain why the presentation of pathology was sporadic in calves [28].

Moreover, we found a statistically significant association between cattle breed and the presence of antibodies against BLV (p≤0.005) (Table-1). These data disagree with the findings published by Betancur and Rodas [15] and Bautista *et al*. [18], who found no significant association. In contrast, the results of this study agree with those of Hernandez *et al*. [27], who also found a statistically significant association between breed and seropositivity using ELISA, showing that breed is a contributing factor for viral infection. Similarly, we determined that the Normando breed and cross-breeds were a preventive factor for BLV infection, whereas the Holstein breed was a risk factor for BLV infection. This can be due primarily to the infection rates of BLV being higher in milk-producing cattle than those in cattle raised for meat [29,30]. Furthermore, some studies have shown that some genetic and breed-specific characteristics are associated with resistance and susceptibility to developing persistent lymphocytosis and lymphosarcoma in animals with EBL [31,32].

We established a statistically significant association between cattle age and seropositivity for BLV (p≤0.05). However, these results do concur with the findings reported by Hernandez *et al*. [27], who were not able to observe such an association. In Sotaquirá, we found that the age range of 1-2 years is considered a protective factor against BLV infection, whereas ages <1 year and more than 4 years were risk factors for the disease. This is due to the disease being present primarily in bovine adults. Therefore, factors, such as the status of the host’s immune system, the infection time of the virus, and the pro-viral load, are important for the pathogenesis of EBL in early age [28].

We further found that the number of births and the age of the cattle is strongly related. Keeping this in mind, it has been reported, particularly in milk cattle, that this parity has a significant effect on infection rates and pro-viral load of BLV. Both factors were elevated in cattle with more descendants, explaining the higher infection rates among milk-producing cows [23].

Moreover, we established a statistically significant association between gender and the presence of antibodies against BLV in the cattle under study. However, these findings disagree with those reported by Betancur and Rodas [15] and Hernandez *et al*. [27], who found no statistically significant association between BLV infection and gender. In contrast, recent studies have shown that milk-producing cows infected with BLV have an altered immune system leading to the deterioration of B and T lymphocytes, affecting general immunity leading to predisposition to infectious diseases [30,33,34]. From this, we conclude that IBR and the presence of mucosal secretion and mastitis are risk factors for BLV presentation in this study.

Similarly, we found artificial insemination to be a risk factor for BLV infection in Sotaquirá. This is likely due to iatrogenic transmission still being the most common transmission method for BLV. This is due to the surgical tools used in the procedure and tubing contaminated with blood during rectal palpation [4]. It has further been established that not changing the tubing during these palpations is associated with a higher number of BLV-positive cattle, turning rectal examination into a potential route of transmission for BLV [2].

Finally, the presence of corrals in the herds under study was established as a risk factor for BLV. This is most likely due to the cattle being submitted to different methods of reproduction and production. Physical contact also drives a significant increase in the pro-viral load, and the infection rate is higher in cows that are permitted close contact with other cattle that takes place in dairy farms where animals are all brought into close contact in holding pens [23].

## Conclusion

A high seroprevalence rate of BLV was found in the municipality of Sotaquirá; this finding is important as it allows for a better estimate of the current state of BLV in the region. This study is a fundamental step in the search for efficient methods for controlling and eradicating the virus. We could determine the key risk factors, such as age, breed, and gender, in the cattle under study. Moreover, some field hand practices were contributing factors. However, the interaction between viral pathogenesis, productive performance, health outcomes, and immune function cannot be fully understood through transversal studies. As such, this study recognizes the need for longitudinal studies that allow for tracking of the animals under study during an extended period.

## Authors’ Contributions

DMBC, DJGC, and DOO: Performed formal analysis and data curation. DMBC, AMD, DJGC, and MOPM: Wrote original draft and reviewed final version of the manuscript. JCTT, DOO, and MOPM: Reviewed the final version of the manuscript. DOO: Designed the study and collected the data. All authors read and approved the final manuscript.

## References

[1] Kobayashi T, Inagaki Y, Ohnuki N, Sato R, Murakami S, Imakawa K (2019). Increasing Bovine leukemia virus (BLV) proviral load is a risk factor for progression of Enzootic bovine leucosis:A prospective study in Japan. Prev. Vet. Med.

[2] Nekouei O, VanLeeuwen J, Sanchez J, Kelton D, Tiwari A, Keefe G (2015). Herd-level risk factors for infection with bovine leukemia virus in Canadian dairy herds. Prev. Vet. Med.

[3] Murakami H, Uchiyama J, Suzukim C, Nikaido S, Shibuya K, Sato R, Maeda Y, Tomioka M, Takeshima S, Kato H, Sakaguchi H, Aida Y, Tsukamoto K (2018). Variations in the viral genome and biological properties of bovine leukemia virus wild-type strains. Virus Res.

[4] Gutiérrez G, Rodríguez S.M, De Brogniez A, Gillet N, Golime R, Burny A, Jaworski J.P, Alvarez I, Vagnoni L, Trono K, Willems L (2014). Vaccination against d-retroviruses:The bovine leukemia virus paradigm. Viruses.

[5] Maresca C, Costarelli S, Dettori A, Felici A, Iscaro C, Feliziani F (2015). Enzootic bovine leukosis:Report of eradication and surveillance measures in Italy over an 8-year period (2005-2012). Prev. Vet. Med.

[6] World Organization for Animal Health (2014). Enzootic bovine leucosis. In:Manual of Diagnostic Tests and Vaccines for Terrestrial Animals. http://www.oie.int/wahis_2/public/wahid.php/Diseaseinformation/statuslist.

[7] Polat M, Takeshima S, Aida Y (2017). Epidemiology and genetic diversity of bovine leukemia virus. Virol. J.

[8] Bartlett P.C, Norby B, Byrem T.M, Parmelee A, Ledergerber J.T, Erskine R.J (2013). Bovine leukemia virus and cow longevity in Michigan dairy herds. J. Dairy Sci.

[9] Corredor-Figueroa A.P, Salas S, Olaya-Galán N.N, Quintero J.S, Fajardo Á, Soñora M, Moreno P, Cristina J, Sánchez A, Tobón J, Ortiz D, Gutiérrez M.F (2020). Prevalence and molecular epidemiology of bovine leukemia virus in Colombian cattle. Infect. Genet. Evol.

[10] Tsutsui T, Kobayashi S, Hayama Y, Yamamoto T (2016). Fraction of bovine leukemia virus-infected dairy cattle developing enzootic bovine leukosis. Prev. Vet. Med.

[11] Pulido-Medellín M, Gonzáñez Ariza W, Bayona H, Chavarro Tulcán G (2017). Determinación de leucosis enzoótica bovina mediante las claves hematológicas de göttingen y elisa en Boyacá, Colombia. Rev. Fac. Cienc. Vet..

[12] Alcaldía Municipal de Sotaquirá (2020). Plan de Ordenamiento Territorial del Municipio de Sotaquirá2008-2011. Consejo Municipal de Sotaquirá. http://www.sotaquira-boyaca.gov.co.

[13] Instituto Colombiano Agropecuario (2019). Tabla de Población Bovina Por Municipio y Departamento. https://www.ica.gov.co/areas/pecuaria/servicios/epidemiologia-veterinaria/censos-2016/censo-2018.aspx.

[14] Figueiredo Marques G, Augusto Pompei J.C, Martini M (2017). Extracción de sangre con sistema de vacío. In:Manual Veterinario de Toma y Envío de Muestras.

[15] Betancur H.C, Rodas G.J (2008). Seroprevalencia del virus de la leucosis viral bovina en animales con trastornos reproductivos de Montería. Rev. MVZ Córdoba.

[16] Carrero Rojas J.L, Arévalo Martínez F, Tarazona Suárez A, Cepeda B.M (2009). Prevalencia de la seropositividad a la leucosis bovina mediante la técnica diagnóstica de ELISA indirecta en hatos lecheros situados en Mesa de los Santos, Santander. Spei Domus.

[17] Benavides Benavides B, Cedeño Quevedo D.A, Serrano de La Cruz M.F (2013). Epidemiological study of bovine leukemia virus in dairy cows in six herds in the municipality of Pasto, Nariño. Rev. Lasallista Investig..

[18] Bautista R, Nova R.Y.A, Pulido-Medellín M.O, Andrade-Becerra R.J (2013). Determinación serológica de leucosis bovina enzoótica en novillas de levante y vacas adultas de la vereda Morichal, Yopal, Casanare. Cien. Y Agric..

[19] Úsuga-Monroy C, Echeverri J, López-Herrera H (2015). Diagnóstico molecular del virus de leucosis bovina en una población de vacas Holstein, Colombia. Arch. Zootec.

[20] Meza-Barreto G, Sanjuanelo-Corredor D.W, Gallego-Marín M.I (2016). Detección molecular del virus de la leucosis bovina:Un estudio por conglomerados en Colombia. Cien. Y Agric..

[21] Gutiérrez G, Alvarez I, Politzki R, Lomónaco M, Dus Santos M.J, Rondelli F, Fondevila N, Trono K (2011). Natural progression of Bovine leukemia virus infection in argentinean dairy cattle. Vet. Microbiol.

[22] Furtado A, Rosadilla D, Franco G, Piaggio J, Puentes R (2013). Leucosis bovina enzoótica en cuencas lecheras de productores familiares del Uruguay. Veterinaria (Montev).

[23] Ohno A, Takeshima S.N, Matsumoto Y, Aida Y (2015). Risk factors associated with increased bovine leukemia virus proviral load in infected cattle in Japan from 2012 to 2014. Virus Res.

[24] Bonifas N, Ulcuango F (2015). Prevalencia de leucosis bovina en la comunidad santo domingo N^o^1, cayambe-ecuador 2012. Rev Cien. Vida.

[25] Sandoval M.R, Delgado C.A, Ruiz G.L, Ramos C.O (2015). Determinación de la seroprevalencia del virus de la leucemia bovina en Lima, Perú. Rev. Investig. Vet. Perú.

[26] Norby B, Bartlett P.C, Byrem T.M, Erskine R.J (2016). Effect of infection with bovine leukemia virus on milk production in Michigan dairy cows. J. Dairy Sci.

[27] Hernandez D, Muñoz J, Alvarez L (2016). Dinámica de la leucosis bovina en el ganado criollo Hartón del Valle en infección natural. Arch. Zoot.

[28] Oguma K, Suzuki M, Sentsui H (2017). Enzootic bovine leukosis in a two-month-old calf. Virus Res.

[29] Murakami K, Kobayashi S, Konishi M, Kameyama K, Yamamoto T, Tsutsui T (2011). The recent prevalence of bovine leukemia virus (BLV) infection among Japanese cattle. Vet. Microbiol.

[30] Andreolla A.P, Scheer Erpen L.M, Frandoloso R, Kreutz L.C (2018). Development of an indirect ELISA based on recombinant capsid protein to detect antibodies to bovine leukemia virus. Braz. J. Microbiol.

[31] Rodríguez S.M, Florins A, Gillet N, de Brogniez A, Sánchez-Alcaraz M.T, Boxus M, Boulanger F, Gutiérrez G, Trono K, Alvarez I, Vagnoni L, Willems L (2011). Preventive and therapeutic strategies for bovine leukemia virus:Lessons for HTLV. Viruses.

[32] Juliarena M, Poli M, Sala L, Ceriani C, Gutierrez S, Dolcini G, Rodríguez E.M, Mariño B, Rodríguez-Dubra E.N (2008). Association of BLV infection profiles with alleles of the BoLA-DRB3.2 gene. Anim. Genet.

[33] Frie M.C, Coussens P.M (2015). Veterinary immunology and immunopathology bovine leukemia virus:A major silent threat to proper immune responses in cattle. Vet. Immunol. Immunopathol.

[34] Ruggiero V.J, Norby B, Benitez O.J, Hutchinson H, Sporer K.R.B, Droscha C, Swenson C.L, Bartlett P.C (2019). Controlling bovine leukemia virus in dairy herds by identifying and removing cows with the highest proviral load and lymphocyte counts. J. Dairy Sci.

